# Sjogren's Syndrome in the Mask of Hypokalemia: A Case Report

**DOI:** 10.31729/jnma.8836

**Published:** 2024-12-31

**Authors:** Krishna Bahadur Sodari, Pragyan Basnet, Krishna Adhikari

**Affiliations:** 1 Achham District Hospital, Mangalsen, Achham, Nepal; 2 Bodegaun Primary Hospital, Indrawati, Sindhupalchok, Nepal; 3 Department of Internal Medicine, Patan Academy of Health Sciences, Lagankhel, Lalitpur, Nepal

**Keywords:** *case report*, *hypokalemia*, *renal tubular acidosis*, *Sjogren syndrome*

## Abstract

Sjogren's syndrome is an autoimmune disease characterized by lymphocytic infiltration of exocrine glands resulting in xerostomia and dry eyes affecting entire body. A 40-year-old woman presented with generalized weakness and shortness of breath. She had reduced muscle power (2/5) with reduced tone, and absent reflexes in four limbs. Further investigation revealed severe hypokalemia with metabolic acidosis. She was treated with intravenous potassium chloride and diagnosed with Sjogren's syndrome based on her serology profile. After treatment, her condition improved. Hypokalemia occurs due to increased renal excretions of potassium. Distal Renal Tubular Acidosis is a major cause of potassium loss in urine. Sjogren's Syndrome is an important cause of Distal Renal Tubular Acidosis, which can present as severe hypokalemia. Although it commonly presents with symptoms like dry eyes and dry mouth, it can rarely present as Distal Renal Tubular Acidosis and hypokalemia as the primary presentation. Sjogren's syndrome should be suspected in patients with hypokalemia with features of Distal Renal Tubular Acidosis.

## INTRODUCTION

Sjgren's syndrome (SS) is an autoimmune disease which is nine times more common in females than in males.^1^ Its rare systemic manifestation is renal involvement which can occur even before the onset of sicca symptoms.^2^ Renal involvement leads to tubular dysfunction causing severe hypokalemia, also seen in several autoimmune diseases like systemic lupus erythematosus and hashimoto thyroiditis.^3,4^ Distal renal tubular acidosis (dRTA) is characterized by inability to acidify the urine maximally (to <pH 5.5) in the presence of systemic acidosis which is found in 25% patients.^5,6^ We report a case of Sjogren syndrome with hypokalemic periodic paralysis due to dRTA, which was initially treated as the case of Systemic Lupus Erythematosus (SLE).

## CASE PRESENTATION

A 40 years old female presented with complaints of generalized weakness for 5 days bilaterally in upper and lower limbs. She complained shortness of breath and generalized abdominal pain for 1 day. The generalized weakness was gradual in onset and slowly progressive. It was symmetrical with difficulty in doing daily activities like combing hair, climbing stairs, standing for long periods and gripping objects. There was no myalgia and arthralgia with no signs of muscle atrophy. The shortness of breath was sudden in onset with absence of fever, cough, abnormal breathing, palpitation and chest pain. Generalized abdominal pain was sudden onset, burning in nature, non-radiating, relieved on rest and exacerbated on exertion. It was associated with nausea, odynophagia, constipation and dizziness. Severity of pain was 6 out of 10 in numerical pain scale.

In the past she was managed as SLE for 2 years on the basis of Anti Neutrophilic Antibody (ANA) positive status at the previous health care center. She was taking tablet hydrocortisone 10 mg once a day and tablet hydroxychloroquine 200 mg twice a day for treatment of SLE. Along with that she had a history of hypokalemia on multiple episodes for which she was treated with potassium chloride infusion on each episode due to unidentified cause. Though signs and symptoms were improved for a short period, they tend to recur over time. The symptoms of generalized muscle weakness gradually worsened over time.

On general examination, she was ill looking, alert and conscious, well oriented to time, place and person. Pallor was present and there were no signs of icterus, cyanosis, clubbing, enlarged lymph nodes and edema. Vital signs were within normal limits except heart rate being 130 beats per minute. On motor examination, power of the muscle was 2/5 on all four limbs, tone was decreased on all four limbs, and tendon reflexes were absent. Her sensory function was intact. On chest examination, decreased bilateral air entry with normal vesicular sound was heard. Normal heart sound was heard on auscultation with no murmurs. On abdominal examination, there was generalized abdominal tenderness and abdominal distension.

Blood investigation revealed severe hypokalemia (serum potassium of 1.3). ECG revealed tachycardia, ST segment depression and U wave which are consistent with severe hypokalemia. After potassium chloride infusion and symptomatic treatment, she was admitted to the intensive care unit for close observation and monitoring. Initially ANA was positive but anti-ds DNA was negative due to which Extractable Nuclear Antigen (ENA) panel was ordered which showed anti-Sjogren's-syndrome-related antigen A (SSA) and anti-Sjogren's-syndrome-related antigen B (SSB) positive and was diagnostic for Sjogren Syndrome.

She was prescribed Injection Methylprednisolone 500 mg once daily, Injection Durataz (piperacillin+tazobactam) 4.5 gram thrice daily, Injection Levofloxacin 500 mg daily, Tablet Methotrexate 7.5 mg once a week, Tablet folic acid 5 mg twice a week, Tablet hydroxychloroquine 200 mg daily, Syrup potassium chloride 15 ml thrice daily and Syrup phosphorus 10 ml thrice daily till last follow up. The generalized weakness of the body improved and power of the all four limbs was 4/5 and bulk, tone, reflexes were normal. Plantar reflex was normal. After improvement in ICU, she was transferred to the medical ward and was treated as a case of SS. She came to follow up after 15 days and no symptoms were present at that time. She was advised to come to next follow up after 3 months.

## DISCUSSION

Hypokalemia is the condition characterized by plasma potassium level <3.5 mmol/L.^7^ Renal symptoms of Sjogren syndrome present in the form of hypokalemia as the first symptoms might create confusion to reach diagnosis in clinical settings.^8^ This case has been reported following the Surgical Case Report (SCARE) guidelines.^9^ It occurs either due to low intake of potassium or increased loss of potassium from the body. The more common increased loss of potassium is due to increased loss through urinary tract or gastrointestinal (GI) tract or inter-cellular shift. GI losses of potassium can result from diarrhea or vomiting and inter-cellular shift of potassium can result from insulin administration, sympathetic overstimulation and thyrotoxicosis. The loss of potassium from urinary tract can occur commonly from primary hyperaldosteronism, cushing's syndrome and renal tubular acidosis (RTA).^10^

Our patient presented with severe hypokalemia and ECG changes. ABG revealed metabolic acidosis with low bicarbonate. These findings were suggestive towards RTA. RTA can be due to primary hereditary causes or secondary causes. SS is one of the important causes of secondary RTA.^11^ SS commonly manifests as xerostomia and dry eyes.^1^ 70-80% of patient's complain of dry mouth with alternating parotid gland swelling and pain in 20% of patients.^11^ The systemic manifestation occurs in 30-40% of patients. Most commonly affected non-exocrine organ is kidney with tubule interstitial nephritis followed by dRTA as the most common renal presentation in SS. The lymphocytic infiltration also causes autoimmune primary biliary cholangitis, and obstructive bronchiolitis etc. Although dRTA is common in Sjogren's syndrome, it is usually asymptomatic and in most cases it remains undetected. Hypokalemia is the most common electrolyte abnormality in patients with dRTA.^8^ Our patient did not have common symptoms of SS as dry mouth and dry eyes rather presented with history of recurrent hypokalemia and signs and symptoms of hypokalemia.

The diagnosis of SS is considered on the basis of classical sign and symptoms but sometimes systemic complication provides the clue to the disease as seen our case.

## CONCLUSIONS

SS is a rare but important cause of severe hypokalemia. It should be suspected in patients with dRTA. It can be misdiagnosed as SLE or other metabolic or rheumatologic disease in patients with severe hypokalemia.
